# Identification of cell senescence molecular subtypes in prediction of the prognosis and immunotherapy of hepatitis B virus-related hepatocellular carcinoma

**DOI:** 10.3389/fimmu.2022.1029872

**Published:** 2022-10-06

**Authors:** Xue Yu, Peng Chen, Wei Yi, Wen Ruan, Xiaoli Xiong

**Affiliations:** ^1^ School of Medicine, Jianghan University, Wuhan, China; ^2^ Department of Integrated Chinese and Western Medicine, Wuhan Children's Hospital (Wuhan Maternal and Child Healthcare Hospital), Tongji Medical College, Huazhong University of Science & Technology, Wuhan, China; ^3^ Department of Respiratory Medicine, Wuhan Children's Hospital (Wuhan Maternal and Child Healthcare Hospital), Tongji Medical College, Huazhong University of Science & Technology, Wuhan, China

**Keywords:** HBV, immune microenvironment, molecular classification, cellular senescent, WGCNA

## Abstract

Hepatitis B virus (HBV)-infected hepatocellular carcinoma (HCC) has a high incidence and fatality rate worldwide, being among the most prevalent cancers. The growing body of data indicating cellular senescence (CS) to be a critical factor in hepatocarcinogenesis. The predictive value of CS in HBV-related HCC and its role in the immune microenvironment are unknown. To determine the cellular senescence profile of HBV-related HCC and its role in shaping the immune microenvironment, this study employed a rigorous evaluation of multiple datasets encompassing 793 HBV-related HCC samples. Two novel distinct CS subtypes were first identified by nonnegative matrix factorization, and we found that the senescence-activated subgroup had the worst prognosis and correlated with cancer progression. C1 and C2 were identified as the senescence-suppressed and senescence-activated subgroups. The immune microenvironment indicated that C2 exhibited a relatively low immune status, higher tumor purity, and lower immune scores and estimated scores, while the C1 subgroup possessed a better prognosis. The CS score signature based on five genes (CENPA, EZH2, G6PD, HDAC1, and PRPF19) was established using univariate Cox regression and the lasso method. ICGC-LIRI and GSE14520 cohorts were used to validate the reliability of the CS scoring system. In addition, we examined the association between the risk score and hallmark pathways through gene set variation analysis and gene set enrichment analysis. The results revealed a high CS score to be associated with the activation of cell senescence-related pathways. The CS score and other clinical features were combined to generate a CS dynamic nomogram with a better predictive capacity for OS at 1, 2, and 3 years than other clinical parameters. Our study demonstrated that cellular senescence patterns play a non-negligible role in shaping the characteristics of the immune microenvironment and profoundly affecting tumor prognosis. The results of this study will help predict patient prognosis more accurately and may assist in development of personalized immunotherapy for HBV-related HCC patients.

## Introduction

Hepatocellular carcinoma (HCC) is the third leading cause of cancer-related fatalities globally, with limited treatment options, high recurrence rates, and poor prognoses. Chronic infection by hepatitis B virus (HBV) is the main cause of hepatocellular carcinoma (HCC) worldwide, which threatens human health and quality of life (1–3). As an increasing number of HBV-related HCCs are diagnosed early, treatment efficacy has significantly improved (4). However, robust prognostic signatures of HBV-related HCC remain unavailable. Moreover, patients with similar tumor stages or pathologic structures may have notably different prognoses, owing to genetic heterogeneity (5, 6). Therefore, it is essential to explore novel and reliable signatures for the precise prognostic prediction of HBV-related HCC. Cellular senescence is a permanent state of cell cycle arrest and is an essential hallmark of malignancy (7). HBV has been linked to telomere shortening (8), leading to hepatocellular senescence and eventually decompensated cirrhosis (9, 10). Cellular senescence displays dichotomous behavior during tumor progression. On the one hand, senescence is an effective barrier to stopping cell proliferation and preventing cells from transforming into infinitely proliferating tumor cells (11), as well as to propagation of ontogenetically activated cells (12). In contrast, the senescent microenvironment secretes pro-inflammatory components due to deficient immune surveillance, thereby generating a pro-tumorigenic milieu that stimulates cancer genesis (13).

There were accumulating evidence suggestting that senescence reprogramming is linked to the mediation of cancer progression (14), metastasis (15), and formulation of the immune microenvironment (16). Through a procedure termed senescence-associated secretory phenotype (SASP), which occurs outside the cell, senescent tumor cells possess the ability to remodel the tumor microenvironment (TME) (17). Paracrine secretion of pro-inflammatory cytokines, chemokines, growth factors, and proteases (e.g., IL-6, IL-8, and TGF-β) by senescent cells in the TME *via* SASP boosts immune surveillance. It promotes tumor immune clearance by transforming the surrounding non-senescent cells into senescent cells. In contrast, maladaptive senescence increases the number of senescent tumor cells to attract and activate myeloid derived suppressor cells (MDSCs) and M2 macrophages *via* SASP, impairing senescent tumor cell clearance and secreting pro-angiogenic substances to enhance vascularization (18, 19). However, the role and mechanism of cellular senescence in the progression and TME formation of HBV-related HCC remains unclear.

In this study, we first identified two CS-based clusters in a cohort of patients *via* the nonnegative matrix factorization(NMF) algorithm. By delineating the two CS-based molecular clusters, we found that they were characterized by diverse clinical features, immune microenvironment characteristics, and biological molecular pathways. The immune microenvironment indicated that C2 senescence-activated clusters had a relatively low immune status and higher tumor purity and presented lower immune scores and estimated scores, while the C1 senescence-suppressed subgroup had a better prognosis. Importantly, we established a novel CS scoring system based on the expression patterns of five CSRGs. It is applicable for predicting the prognosis and immune environment of patients with HBV-related HCC.

## Materials and methods

### Preprocessing of hepatocellular carcinoma dataset and download of cellular senescence genes

The Cancer Genome Atlas (TCGA; https://portal.gdc.cancer.gov/), Gene Expression Omnibus (GEO; https://www.ncbi.nlm.nih.gov/geo/), and International Cancer Genome Consortium (ICGC; https://dcc.icgc.org/) were used to procure and file gene expression profiles of patients with HBV-related HCC and combined detailed clinical cohort information. In the current study, we included three independent liver cancer cohorts for the analysis (TCGA-HCC, GSE14520, and ICGC-LIRI). The clinical baseline characteristics of the three datasets are illustrated in **Table 1**. We consulted the CellAge database (https://genomics.senescence.info/cells/index.html), which comprises 279 experimentally validated cellular senescence genes. In HBV-related HCC cohorts, we used the ComBat package (20) to correct batch effects from different datasets.

**Table 1:** Baseline characteristics of patients in the training and two validation datasets.

	Level	Training dataset	Validation datasets	
		TCGA-LIHC, *n *= 355	GSE14520, *n *= 203	ICGC-LIRI, *n *= 243
Sex	Male	240 (67.6)	175 (86.2)	182 (74.9)
	Female	115 (32.4)	28 (13.8)	61 (25.1)
Age in years	≤50	73 (20.6)	100 (49.3)	17 (7.0)
	>50	282 (79.4)	103 (50.7)	226 (93.0)
HBV infection	No	22 (6.2)	6 (3.0)	/
	Yes	134 (37.7)	195 (96.0)	/
	Unknown	199 (56.1)	2 (1.0)	/
HCV infection	No	57 (16.1)	/	/
	Yes	99 (27.9)	/	/
	Unknown	199 (56.1)	/	/
Alcohol consumption	No	232 (65.4)	/	/
	Yes	113 (31.8)	/	/
	Unknown	10 (2.8)	/	/
Cirrhosis	No	/	16 (7.9)	/
	Yes	/	187 (92.1)	/
Child-Pugh stage	A	211 (59.4)	/	/
	B/C	22 (6.2)	/	/
	Unknown	122 (34.4)	/	/
AFP in ng/mL	≤300	206 (58.0)	108 (53.2)	/
	>300	63 (17.7)	92 (45.3)	/
	Unknown	86 (24.2)	3 (1.5)	/
Tumor size in cm	≤5	/	134 (66.0)	/
	>5	/	69 (34.0)	/
Tumor number	Solitary	/	163 (80.3)	/
	Multiple	/	40 (19.7)	/
Edmondson grade	I/II	227 (63.9)	/	158 (65.0)
	III/IV	128 (36.1)	/	65 (26.7)
	Unknown	0	/	20 (8.2)
Vascular invasion	No	199 (56.1)	/	/
	Yes	102 (28.7)	/	/
	Unknown	54 (15.2)	/	/
AJCC stage	I/II	161 (79.3)	263 (74.1)	146 (60.1)
	III/IV	42 (20.7)		97 (39.9)
BCLC stage	A	/	157 (77.3)	/
	B/C	/	46 (22.7)	/

HBV, hepatitis B virus; HCV, hepatitis C virus.

### Univariate cox regression and unsupervised clustering of cell senescent-related subtypes

Before further analysis, samples with less than 30 days of the follow-up period were excluded. First, we performed a univariate Cox approach using the survival package to identify the gene set for cellular senescence associated with prognosis. P < 0.001 in the univariable Cox regression analysis was considered statistically significant. The liver cancer cohort was then precisely grouped using the clustering tool of an unsupervised clustering algorithm. A total of 793 meta-cohorts of HBV-related HCC patients, including three datasets, were utilized to authenticate the HCC cohort senescence subtypes based on the expression profiles of 51 prognosis-related cellular senescence genes as screened by univariate Cox regression. The optimal K value, in which the K value was adopted as the number of subgroups, was chosen at the point where the magnitude of the covariance correlation coefficient started to decrease with an increasing number of optimal clusters (21); thus, two cellular senescence subtypes, C1 and C2, were inscribed. The cancer subtype package performs an unsupervised clustering algorithm (22). Survival analysis of the two subtypes and genes associated with cellular senescence prognosis were visualized by the “survival” and “survminer” R packages (23), in which the differences with p-values less than 0.05 were statistically significant.

### Analysis of the senescent enrichment hallmarks and clinical characteristics in different subtypes

The “clusterProfiler” package in the R software was utilized to undertake gene set enrichment analysis (GSEA), and the HALLMARK gene sets were adopted as the enrichment gene sets (24). Gene set variation analysis (GSVA) was also performed using the R program’s “GSVA” package (25). We then employed the limma package to analyze the differences in the results of different subtypes of GSVA and visualized them in the form of heat maps using the heatmap package. Subsequently, a total of 343 TCGA-HCC cohort patients with clinical information of different subtypes were included for clinical correlation analysis, with C1 and C2 clusters having different clinical characteristics.

### Analysis of the composition of the immune microenvironment in cellular senescence subtypes

To further investigate the differences in tumor immune composition due to the CS process, ESTIMATE (26) and single-sample gene set enrichment analysis (ssGSEA) algorithms (27, 28) were used to evaluate the immune-related scores and functions between CS patterns.

### Derivation and verification of the cellular senescence score

First, we performed further filtering based on the 51 prognosis-related cellular senescence genes that were previously identified as significant. To formulate the CS score model and avoid an over-fitted scenario, the “glmnet” R package was employed for the lasso algorithm, and the following equation was implemented to determine the risk score for each patient (29). CS risk score=∑Gi∗Bi, where Gi is the expression level, and Bi is the coefficient. Depending on the median CS score, the cohort patients were split into a low-risk group and a high-risk groups. Subsequently, a dimensionality reduction investigation between various risk subgroups was undertaken to utilize principal component analysis (PCA) and the nonlinear dimensionality reduction approach (tSNE). Kaplan-Meier survival data were used to investigate the connection between CS scores and OS. A time-independent survival receiver operating characteristic (ROC) curve was also plotted to assess its predictive usefulness. The other two cohorts served as the test and validation datasets, respectively, whereas the TCGA-HCC cohort was used as the training dataset to create the model.

### Construction of the WGCNA to detect the modules correlated to cellular senescent properties

The underlying co-expression modules positively linked with the high- and low-risk groups were uncovered by generating a weighted gene co-expression network analysis using the WGCNA program (30), which further revealed molecular functional differences between the high- and low-risk groups. The soft thresholds for the scale-free networks were determined as β=14. The similarity of the topological overlap matrices was evaluated to assess the distance between gene modules. Furthermore, we regarded the CS score as a clinical factor and examined the relationship between the different gene modules. Spearman correlation coefficients between high- and low-risk traits and functional modules were dominated by the correlation index and matching p values. Gene significance (GS) and module membership (MM) were computed for each module. After selecting the modules that were most closely related to the high- and low-risk groups, gene ontology (GO) and Kyoto encyclopedia of genes and genomes (KEGG) analyses were performed on the chosen modules.

### Correlation analysis of the CS score and immune regulation

To measure the immunity landscape between different risk groups, seven algorithms (CIBERSORT, MCPcounter, QUANTISEQ, XCELL, CIBERSORT-ABS, EPIC, and TIMER) from TIMER 2.0 (31) (http://timer.cistrome.org/) were implemented. Therefore, correlation and immune infiltration qualification evaluations were used to evaluate immune infiltration between distinct CS groups. The immune checkpoint expression matrix was extracted and incorporated into subsequent correlation investigations.

### Establishment and validation of the nomogram containing senescence risk coefficients

The CS risk score was assessed using univariate and multivariate Cox regression models to determine whether it is a reliable prognostic indicator for HBV-related HCC populations. Additionally, we used the “RMS” R package to create a dynamic nomogram model with CS risk score parameters for overall survival forecasting. The generated nomogram included two factors for the risk score and variables for the clinical stage. We also presented the calibration curve, the 45° line reflecting the best prediction, to further assess the accuracy of the nomogram plot. The consistency index (C-index) between actual and projected probabilities was used to calculate the predictive capacity of the dynamic Norman plot. Furthermore, we tested the prognostic diagnostic effectiveness of the dynamic Norman diagram using time-dependent ROC analyses (1, 2, and 3 years). Moreover, we performed decision curve analysis (DCA) using the R package “rmda” (32) to examine the clinical value of the dynamic nomogram.

### Statistical analysis

In this investigation, categorical and quantitative data were juxtaposed using chi-square and Mann-Whitney U tests. Visual and statistical comparisons were performed using R version 4.0.2. Differences between the high- and low-risk groups were tested using the Mann–Whitney test for non-normally distributed variables and the unpaired t-test for normally distributed variables.R software (version 4.0.3) was used for all statistical analyses, and a two-sided P value of 0.05 was set as the threshold for significance.

## Result

### Identification of cell senescence gene-based subtypes in the combined meta-data cohort

**Figure 1:**
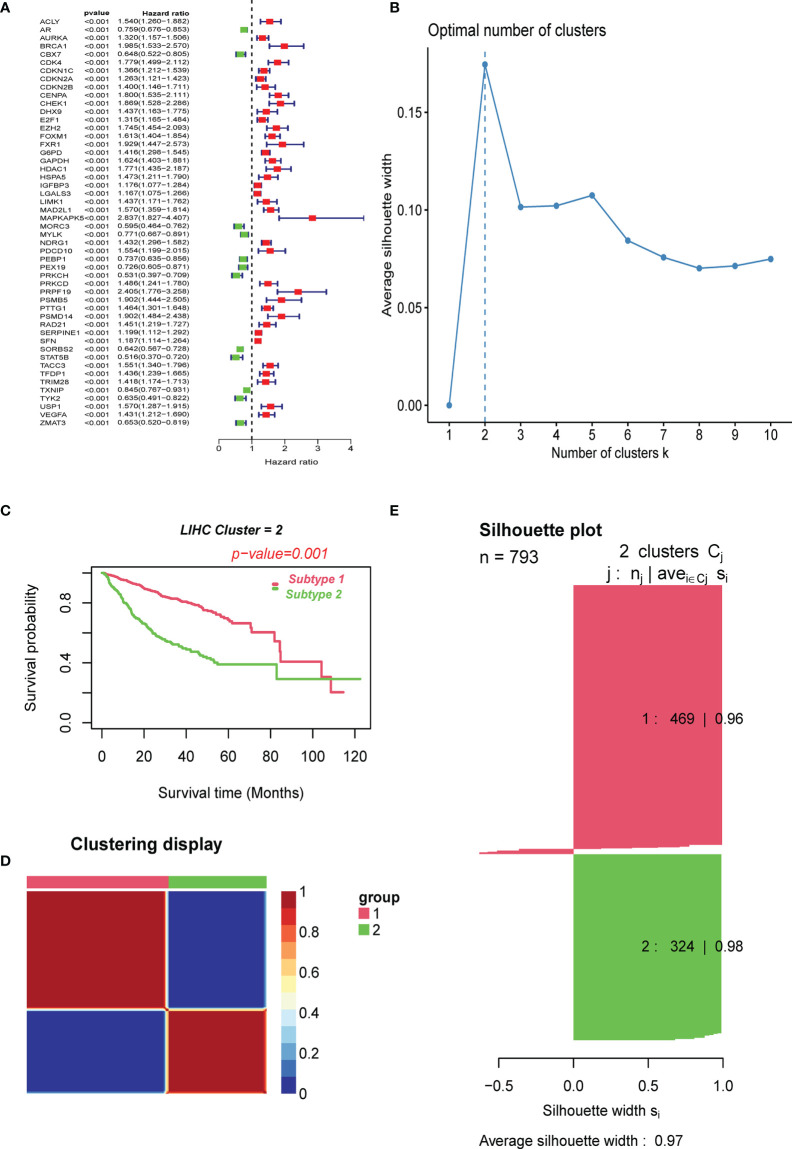
Clustering of cell senescence genes–based subtypes in combined meta-data cohorts using NMF algorithm. **(A)** Screening of prognostic CSRGs by univariate Cox regression analysis. **(B)** The optimal cluster number was determined to be two. **(C)** The log-rank test P-value for survival curve analysis of the patients in the two clusters. **(D)** The clustering heat map visualizes the degree of segmentation in sample clustering. **(E)** The average silhouette width represents the coherence of clusters.

We first performed a univariate Cox analysis using the relevant cellular senescence gene expression profiles from TCGA and clinical data to identify 51 cellular senescence-associated genes affecting the overall survival time in liver cancer (**Figure 1A**). Among the 51 prognosis-related CS genes, 12 had HR values less than 1, and most of the others were risk factors with HR values >1. After correcting for batch effects, we performed an unsupervised pattern analysis on the combined metadata cohort (TCGA-HCC, GSE14520, and ICGC-LIRI) that consisted of data of all 793 samples to better comprehend the cellular senescence patterns in HBV-related HCC. The ConsensusClusterPlus package was then used to evaluate the stability of the clusters based on the 51 prognosis-related genes previously screened, and we observed that two clusters exhibited the ideal number. (**Figures 1B, D, E**). We then performed unsupervised consensus clustering on the above cohort samples to identify CS subtypes, further PCA analyses of which revealed distinct discrimination between the two phenotypes (**Supplementary Figure 1**). We termed these cellular senescence-suppressed (C1) and senescence-activated (C2). Furthermore, overall survival (OS) analysis revealed that the C1 cluster had a much better prognosis than the C2 cluster (P<0.001, log-rank test; **Figure 1C**).

### GSVA analysis of the CS-enrichment pathway hallmarks

**Figure 2:**
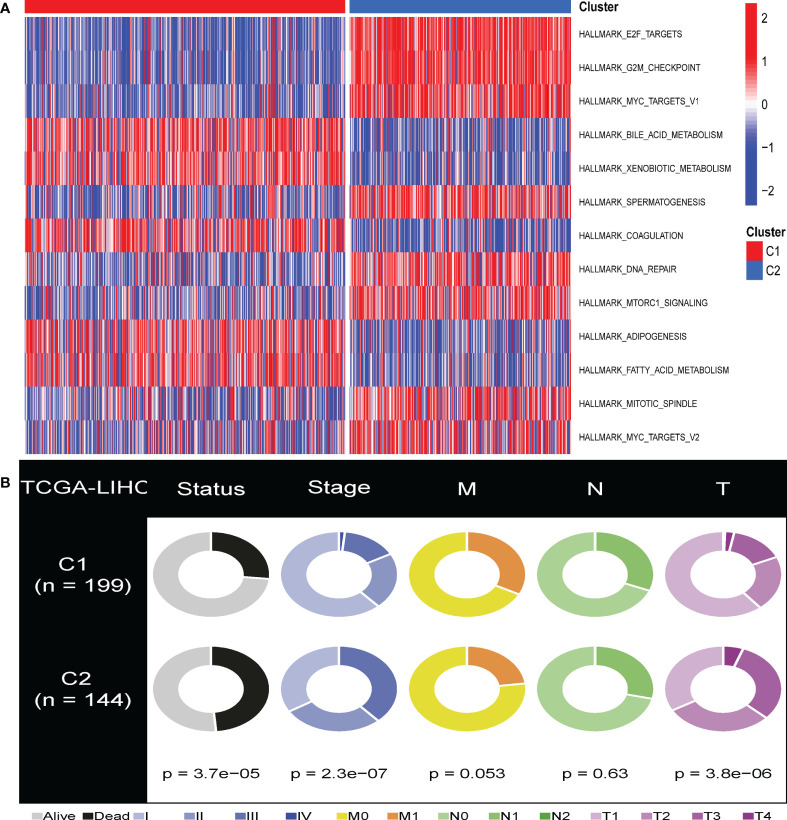
GSVA analysis of the senescent-enrichment pathway hallmarks and clinical characteristics in distinct CS patterns. **(A)** The differences between the C1 and C2 subtypes in the enriched hallmark pathways. **(B)** Pie charts illustrated the different distribution of clinical characteristics between the two subtypes between C1 and C2 subtypes in TCGA-HCC cohorts.

Furthermore, to investigate the link between enriched pathways and the survival of HBV-related HCC CS patterns, we utilized GSVA analysis to elucidate the discrepancies between enrichment pathways (**Figure 2A**). GSVA results showed that many differentially expressed pathways were enriched among subtypes, which were then visualized using a heat map. Compared with cluster 1, pathways related to cell cycle regulation, G2M checkpoint, DNA damage repair, and spindle formation were significantly overexpressed in cluster 2. In addition, the C2 subclass was significantly enriched in the E2F transcription factor pathway and the C-MYC target gene pathway relevant to tumor progression. In contrast, adipogenesis, bile acid metabolism, and fatty acid metabolism pathways related to lipid metabolism were significantly downregulated. This is one of the reasons why the C2 subtype has a worse prognosis than the C1 subtype. This phenomenon was consistent with previous reports that identified the manifestation of senescent subtypes of clear cell renal cell carcinoma (33).

### Association of cellular senescence patterns with clinical characteristics of HBV-related HCC Patients

We compared the various clinical features of different patterns to determine the role of cellular senescence in advancing HBV-related HCC. We found that C1 and C2 had significant differences in survival, T stage, and stage features (**Figure 2B**, P<0.05). This result indicated that cellular senescence patterns might be attributed to the prognosis and progression of HBV-related HCC.

### Cellular senescent subpopulations exhibit different tumor immune function characteristics

**Figure 3:**
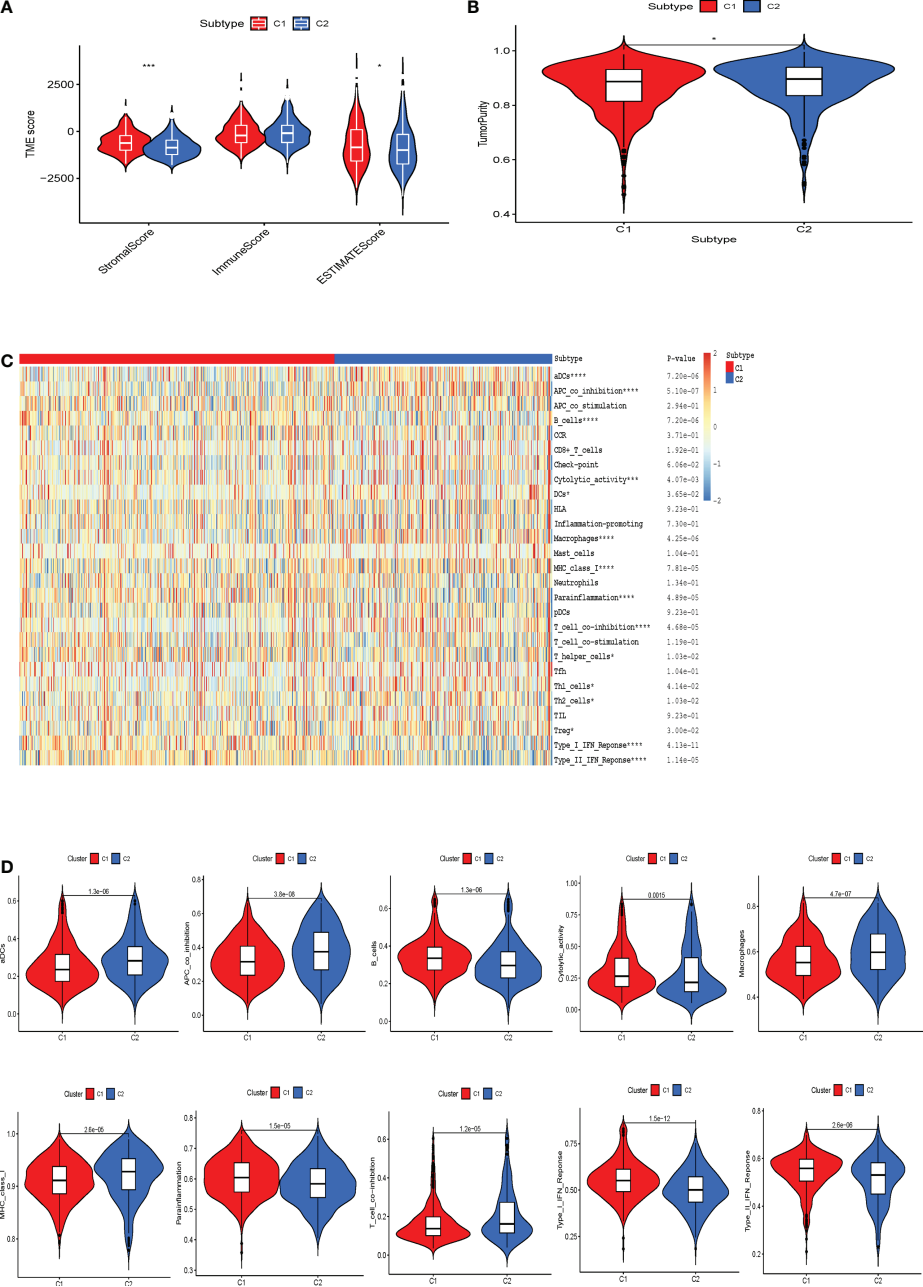
The correlation between the tumor microenvironment features and the CS patterns. **(A)** Quantitative comparison of estimated, immune and stromal scores of the two phenotypes. **(B)** Quantitative comparison of the tumor purity of the two phenotypes. **(C)** Visualization of the two CS phenotypes by heat map to obtain the enrichment levels of 29 immune-related cells as assessed by the ssGSEA algorithm and quantitative analysis. *p < 0.05; ***p < 0.001; ****p < 0.0001. **(D)** Box plots exhibited the degree of difference in immune composition between the two phenotypes.

Next, we performed immunological analysis of both subtypes. The estimation algorithm revealed that the stromal score, as well as the estimated score, was higher in subtype C1 than in subtype C2. At the same time, tumor purity was higher in C2 than in C1, and no significant differences were found between the two immune scores (**Figures 3A, B**). The infiltration of immune cells into various cellular senescence subpopulations was estimated using the ssGSEA algorithm. We revealed that compared to cluster 2, cluster 1 had significantly higher levels of para-inflammation, B cells, cytolytic activity, and interferon (IFN) response infiltration. Dendritic cells, APC co-inhibition cells, macrophages, MHC class 1, and T cell co-inhibition were far more prevalent in cluster 2 than in cluster 1 (**Figures 3C, D**). Furthermore, TIDE algorithm was applied to predict the likelihood of immunotherapy response of each CS-based patterns of HBV-related HCC patients (**Supplementary Figure S1**). Based on the TIDE algorithm, C2 was predicted to be much more responsive to immunotherapy than C1, and C1 had a higher TIDE score than the C2 cluster.

### Development of CS score for prediction prognosis

**Figure 4:**
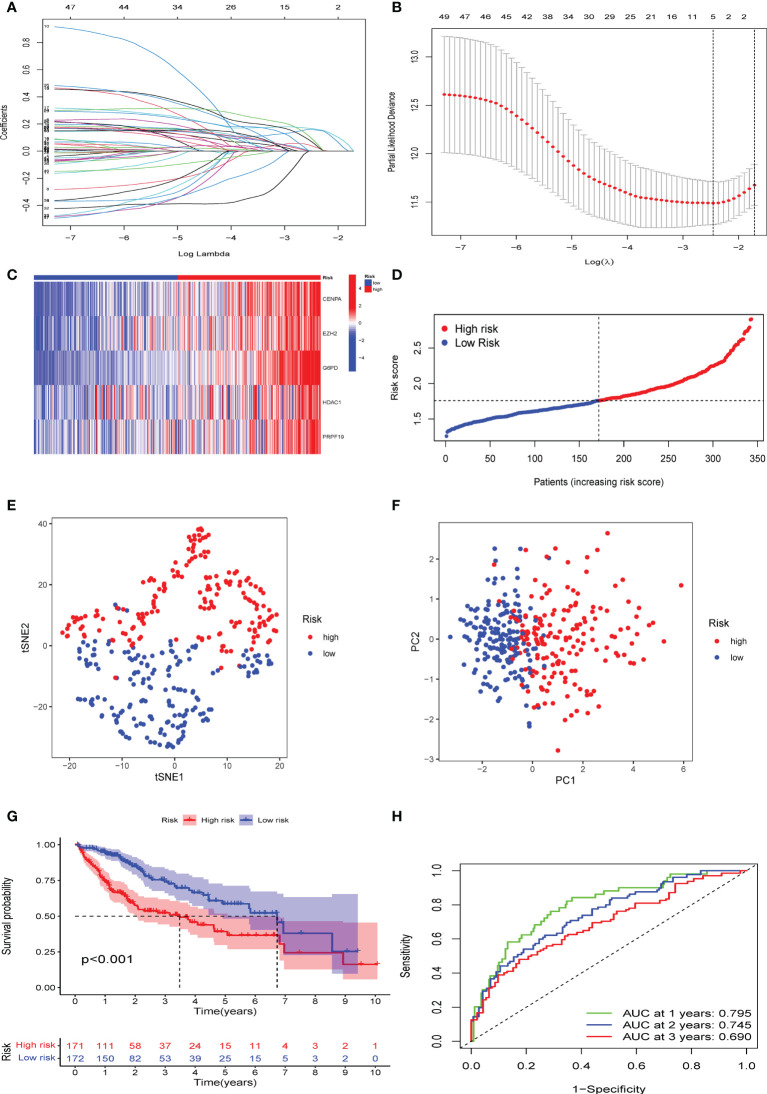
Establishment of a novel CS score signature. **(A)** Identification of the respective regression coefficients of 51 prognostic genes by lasso regression. **(B)** Lasso regression narrowed down the CS genes to create an optimal multivariate signature. **(C)** Heat map visualization of the trend of five candidate genes in the training cohort with CS score. **(D)** The values of CSRG-based risk scores were distributed in each group of patients in the high-risk and low-risk groups. **(E)** PCA and **(F)** t-SNE reduction illustrated the patients in different risk groups were distributed in two directions. **(G)** Survival analysis results of patients of different risk groups in the training cohort. **(H)** Results of time-dependent ROC curves for the risk model in the training cohort.

CS patterns profoundly drive tumor development and reshape the immune microenvironment, resulting in diverse patient prognostic outcomes. Therefore, we constructed a multivariate model containing five CS gene variables (CENPA, EZH2, G6PD, HDAC1, and PRPF19) to predict the overall survival time in different patient cohorts by combining univariate Cox lasso machine learning algorithms (**Figures 4A–D**). Each of the five gene expression levels were multiplied by the corresponding coefficient indices to yield the patient’s individual CS risk scores. According to Kaplan-Meier analysis, these five genes were independent prognostic indicators of HBV-related HCC (**Supplementary Figure S2**). Using the median of the calculated scores for cellular senescence, we divided the patients into two groups with high- and low-risk scores. We observed markedly worse survival in the group with a high senescence risk score (**Figure 4E**). Similarly, PCA and t-SNE evaluations were conducted to determine whether these two risk groups were distributed. CS scores for OS at 1, 3, and 5 years were 0.795, 0.745, and 0.690, respectively, according to time-dependent ROC curve analysis (**Figure 4F**).

### External verification of the CS signature in the GSE14520 and ICGC-LIRI cohorts

**Figure 5:**
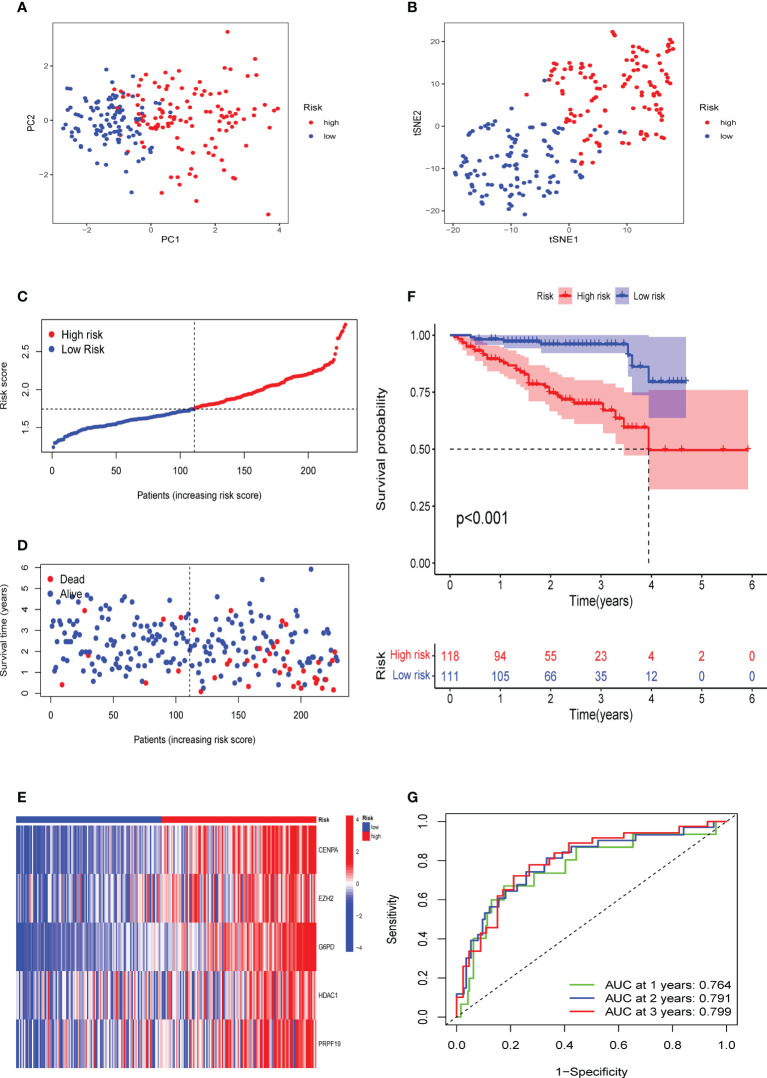
Verification of the CS signature in the testing cohort GSE14520. **(A)** PCA and **(B)** t-SNE analysis of the testing cohort GSE14520. **(C, D)** Patterns of alteration in patient survival status and survival time between the high- and low-risk groups based on the accumulation in CS scores. **(E)** Heat map visualization of the expression trend of 5 CS genes with increasing CS score in the GSE14520 cohort. **(F)** Survival analysis of patients in the high-risk and low-risk groups in the validation cohort testing cohort GSE14520. **(G)** Validation of time-dependent ROC curve results for the risk model in the cohort.

**Figure 6:**
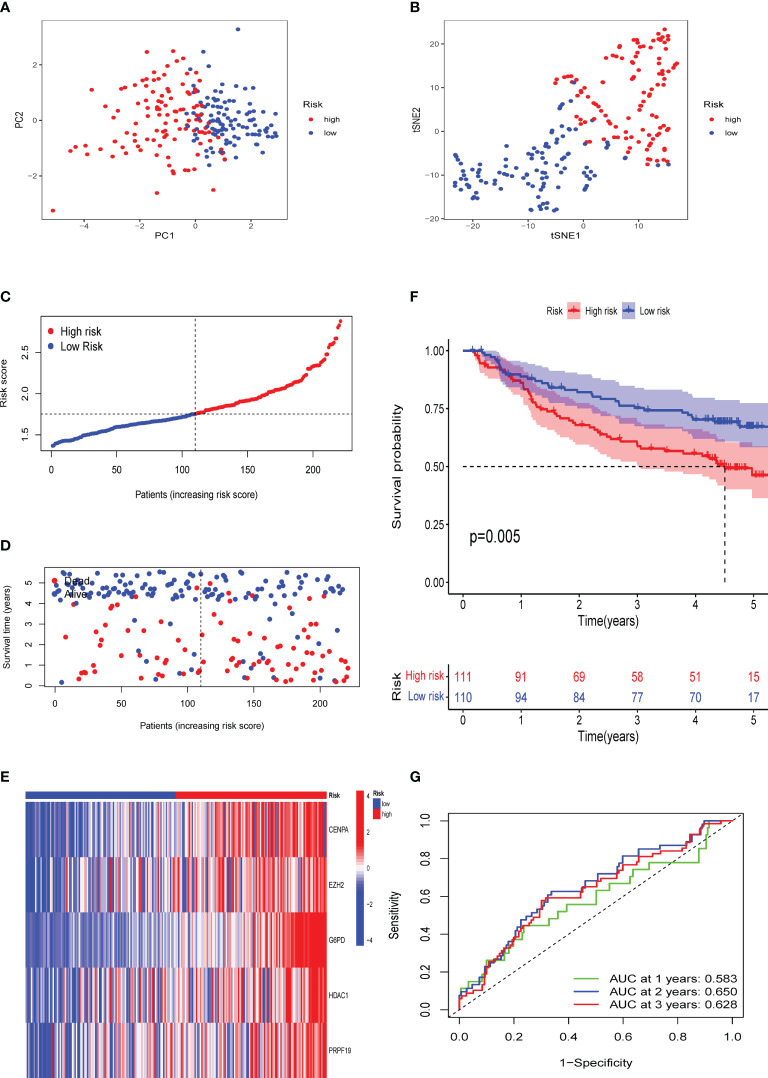
Validation of the CS signature in the verification cohort ICGC-LIRI. **(A)** PCA and **(B)** t-SNE analysis of the ICGC-LIRI cohort. **(C, D)** Patterns of alteration in patient survival status and survival time between the high- and low-risk groups based on the accumulation in CS scores. **(E)** Heat map visualization of the expression trend of 5 CS genes with increasing CS score in the ICGC-LIRI cohort. **(F)** Survival analysis of patients in the high-risk and low-risk groups in the validation cohort ICGC-LIRI. **(G)** Validation of time-dependent ROC curve results for the risk model in the cohort.

To confirm the solid prognostic value of the signature, we further confirmed these conclusions in the testing cohort GSE14520 and verified them in the ICGC-LIRI cohort. As mentioned earlier for the previous training set, the formula was employed to calibrate the CS risk scores in GSE14520 (n=242) and ICGC-LIRI (n=232), and the cohorts were then partitioned into high- and low-risk groups following the training cohort. The PCA and tSNE tests also revealed considerable differences between the high- and low-risk groups in the GSE14520 and ICGC-LIRI cohorts, supporting the earlier findings from the training set (**Figures 5A, B** and **6A, B**). Mortality increased with increasing risk scores (**Figures 5C–E** and **6C–E**), with significantly lower OS in high-risk patients (**Figures 5F** and **6F**). The scoring system in the GSE14520 predicted AUC values for OS at 1, 3, and 5 years as 0.764, 0.791, and 0.799, respectively, whereas the risk score in the ICGC-LIRI predicted AUC values for OS at 1, 3, and 5 years of 0.583, 0.650, and 0.628, respectively (**Figures 5G** and **6G**). Furthermore, we compared this CS signature with those previously reported ones and found it had displayed comparable, or even better in a certain condition, AUCs for OS prediction. Most importantly, this prognostic model demonstrated better reliability since its performance was satisfactory and consistent in two external validation sets (**Table 2**).

**Table 2:** The comparison with previously reported clinical signatures.

References	Model	Training set	AUC (1-, 3-, 5-year OS)	Validation set one	AUC (1-, 3-, 5-year OS)	Validation set two	AUC (1-, 3-, 5-year OS)
CS signature	5-gene	TCGA (*n* = 343)	0.795, 0.745, 0.690	ICGC (*n* = 229)	0.764, 0.791, 0.799	GSE14520 (*n* = 221)	0.583, 0.650, 0.628
Yan *et al.* 2019 (34)	4-gene	TCGA (*n* = 236)	0.72, 0.71,0.61	TCGA (*n* = 118)	0.71, 0.57, 0.55	GSE76427 (*n* = 115)	0.63, 0.66, 0.72
Li *et al.* 2020 (35)	6-gene	TCGA (*n* = 365)	0.76, 0.68, 0.69	ICGC (*n* = 243)	0.68, 0.7, 0.68	/	/
Zhang *et al.* 2020 (36)	14-gene	TCGA (*n* = 312)	0.71, 0.74, 0.64	GSE14520 (*n* = 225)	0.64, 0.59, 0.65	GSE76427 (*n* = 114)	0.60, 0.61, 0.60
Liu *et al.* 2020 (37)	4-gene	TCGA (*n* = 343)	0.70, 0.71, 0.68	GSE14520 (*n* = 215)	0.72, 0.70, 0.68	/	/
Li et al., 2022 (38)	6-gene	TCGA (n = 343)	0.768, 0.691, 0.666	ICGC (*n* = 231)	0.683, 0.701, 0.641	/	/

### WGCNA analysis

**Figure 7:**
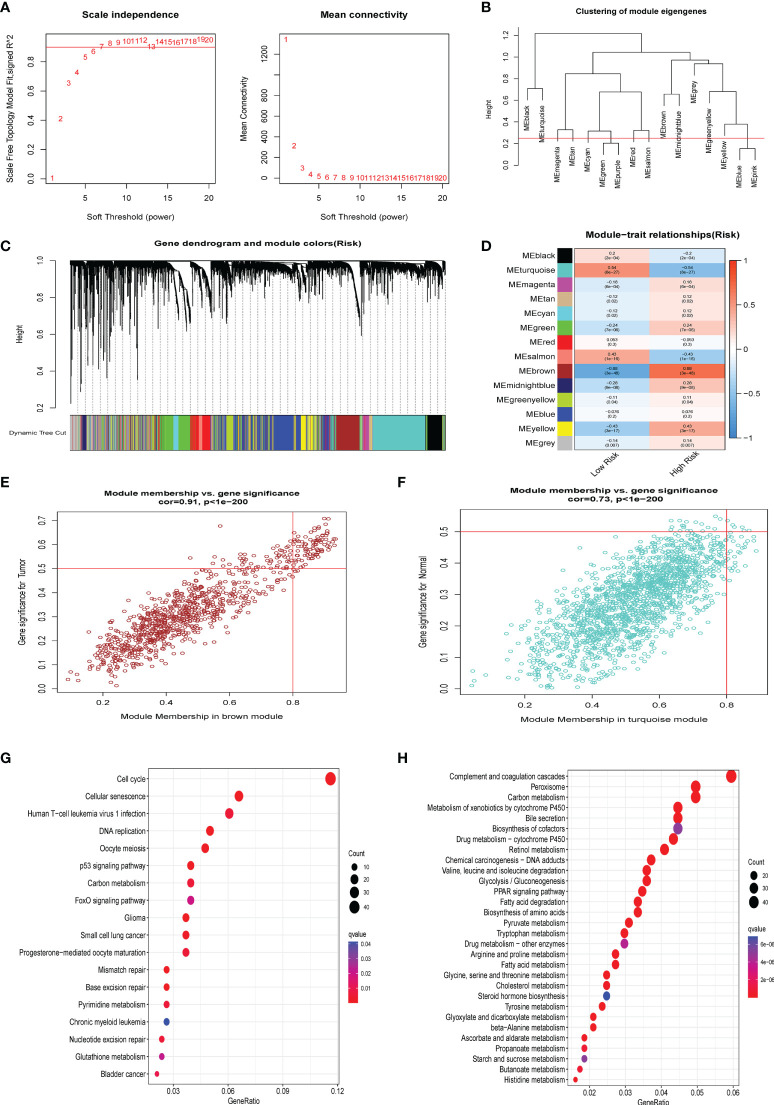
WGCNA analysis. **(A)** The 14 was elected as the soft threshold parameter value to build and fit the scale-free co-expression network. **(B)** Clustering of module eigengenes. **(C)** The branches of the tree diagram correspond to the 14 highly co-expressed gene modules and are color-coded. **(D)** Correlation coefficients and corresponding P-values between each color co-expressed gene module and Risk score are shown in boxes and visualized in different color-block shades. **(E) **The scatter plot presents the correlation between gene memberships in the brown module. **(F) **The scatter plot presents the correlation between gene memberships in the turquoise module. **(G)** The significant KEGG results of the Brown module. **(H)** The significant KEGG results of the Turquoise module.

We generated a novel CS-based model to predict HBV-related HCC survival and TME infiltration in the previous stage. We formed a gene co-expression network using the WGCNA package to identify the modules most vital to the CS risk scores and further investigate the probable biological molecular functions occurring in the high- and low-risk groups. The number 14 was selected as the acceptable soft threshold, from which we constructed a scale-free co-expression network (**Figure 7A**). As a result, 14 gene modules were obtained based on mean hierarchical clustering and dynamic tree clipping filtering (**Figures 7B–D**). The results revealed that the brown module was more associated with the high-risk group (cor=0.68, P< 3^e-48^), whereas the turquoise module was more associated with the low-risk group (cor=0.54, P< 8^e-27^; **Figures 7E–F**). Finally, we filtered the grey and the turquoise modules for further functional GO (**Supplementary Figure S3**) and KEGG (**Figures 7F, G**) enrichment analyses (**Figures 7H**). KEGG enrichment results proved that cell cycle-related pathways were enriched in the brown module, while lipid metabolism-related pathways were dominant in the turquoise module.

Moreover, the correlation heatmap further indicated that the trend of CS scores correlated with multiple cell cycle signaling pathways and oncogenic signatures, consisting of MYC and E2F target gene sets, in contrast to pathways such as lipid metabolism, which were negatively correlated with the CS scores (**Supplementary Figure S3**). Our findings also agree with the current view that CS is accompanied by the cessation of cell division (39) and abnormal activation of oncogenes (40). Our results are consistent with the previously identified GSVA hallmark phenomena in CS patterns. This was further confirmed from another perspective that a CS score can accurately distinguish a patient’s cellular senescent state.

### Correlation analysis of the CS score and immune regulation

**Figure 8:**
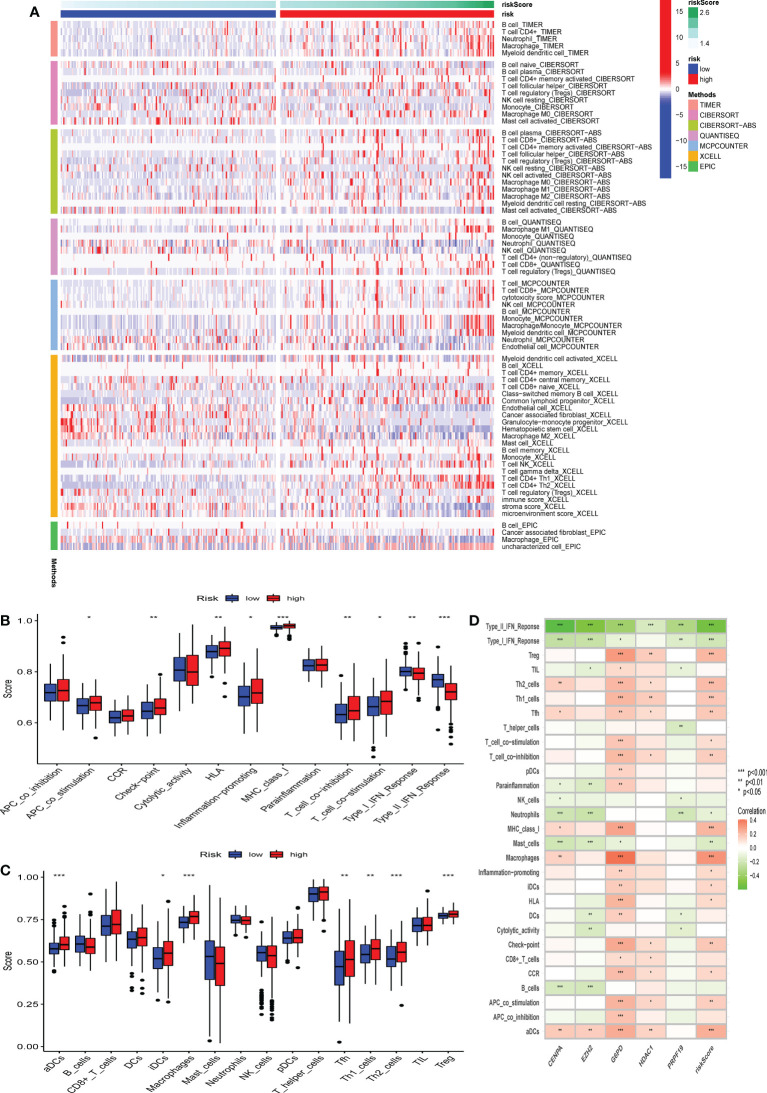
The difference in immune infiltration among patients in different risk groups. **(A)** Seven deconvolution immunization algorithms visualise the immune cell differences between high and low-risk groups. **(B-C)** The box plot illustrated the absolute abundance scores of the 16 immune cells and 13 immune function components in different risk groups. **(D)** The correlation heatmap between the absolute abundance scores of immune cells and the immune function and the CS-score. *Adjusted p < 0.05, ** adjusted p < 0.01, *** adjusted p < 0.001.

**Figure 9:**
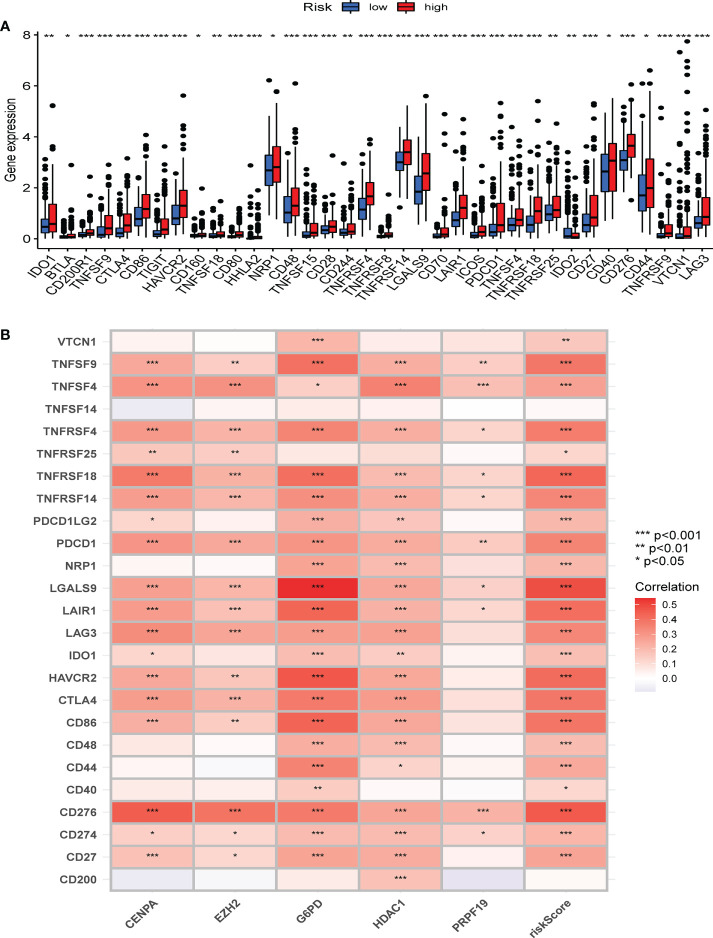
The correlation between immune checkpoint and the CS risk score. **(A) **The expression of the classic immune checkpoint genes between different risk groups. **(B)** The correlation heatmap between classic immune checkpoint genes, the five CS signature genes, and the CS risk score. *Adjusted p < 0.05, ** adjusted p < 0.01, *** adjusted p < 0.001.

To further characterize the influence of the proposed cellular senescence risk score in shaping the immune microenvironment, we applied seven immune deconvolution methods to draw the high- and low-risk immune landscapes (**Figure 8A**). Comparative analysis of immune cells and functional pathways supported the existence of distinct immune cell subsets in the two risk groups for APC co-stimulation: checkpoint, HLA molecules, MHC class I, T cell co-inhibition, T cell co-stimulation, and type I and type II IFN responses, along with B cells, iDCs, mast cells, neutrophils, NK cells, T helper cells, Th1, Th2, and Treg (P<0.05; **Figures 8B, C**). Subsequently, we examined whether immune checkpoint inhibitors were modulated in response to the CS scores. We found significant differences in the expression of the majority of immunological checkpoints between the two risk groups (**Figure 8D**). The prevalence of most immune checkpoint genes showed a significantly positive connection between the five CS signature genes and the CS risk score (**Figure 9A, B**). These results suggest that the risk scores may be related to immunotherapy. A previous study had demonstrated that a higher TIDE score indicated worse immunotherapy response. Therefore, TIDE algorithm was employed to predict the clinical response to anti-PD1 and anti-CTLA4 treatments. The TIDE score in TCGA-HBV-HCC cohorts with high CS risk score was higher than that in the low CS risk score subgroup (**Supplementary Figure S1**).

Overall, our study indicates that the high CS risk score might suggest poor outcome of anti-PD1 and anti-CTLA4 therapy. Meanwhile, CS risk score might be a potential biomarker for evaluating the immunotherapy effect and prognosis in HBV-related HCC.

### Predictive performance of cellular senescence model in clinical application

**Figure 10:**
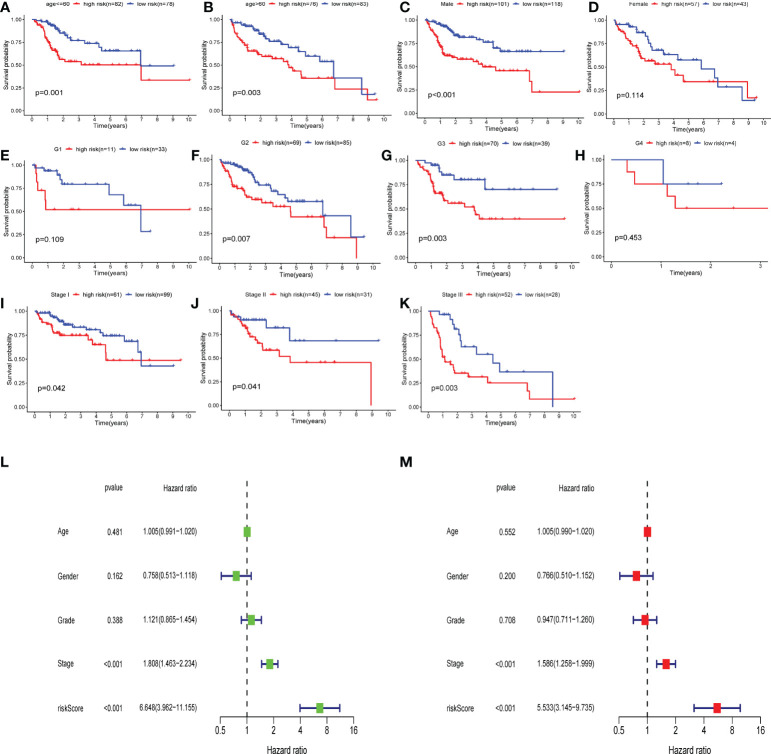
Independent predictive analysis and subgroup analysis of CSRGPI. The subgroup survival analysis was stratified by age **(A, B)**, gender **(C, D)**, grade **(E–H)**, and stage **(I–K)** to further confirm the risk stratification ability of CSRGPI. **(L, M)** Forest plot visualization of univariate and multivariate cox regression results in the TCGA-HCC cohort.

Subsequently, we carried out a subgroup test to evaluate the prognostic significance of the CS score in subgroups of patients with different clinical characteristics, in which clinical features included age (**Figures 10A, B**), gender (**Figures 10C, D**), grade (**Figures 10E-H**), and stage (**Figures 10I-K**). The findings demonstrated that the high-risk group was significantly associated with a worse prognosis in all subgroups except for female patients (**Figure 10D**), G1 (**Figure 10E**), and G4 (**Figure 10H**). Therefore, univariate and multivariate Cox regression analyses were conducted to explore the prospective independence of CSRGS compared with other typical clinicopathological variables. The results demonstrated that the CS-score model had a high predictive ability for patient prognosis in practical applications (**Figures 10L, M**).

### Comparison of the dynamic nomogram to prove the predictive value of the CS score signature

**Figure 11:**
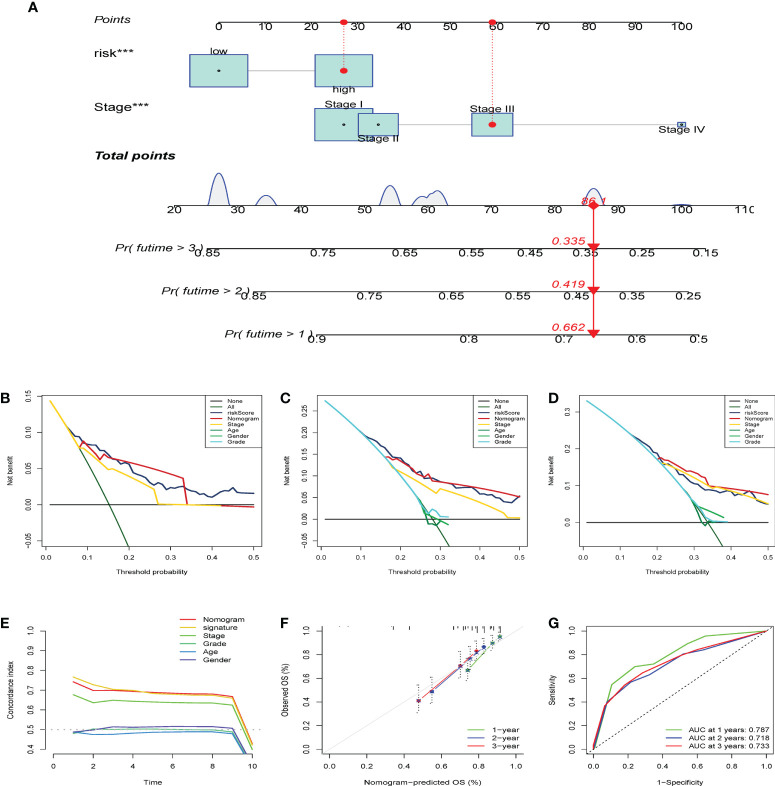
Establish of CS-dynamic nomogram and evaluate its clinical performance and benefits of HBV-related HCC. **(A) **The dynamic nomogram integrates the variables (CS risk scores and stage) for predicting survival status at 1- year, 2-year, and 3-year. **(B–D)** DCA was employed to report the respective clinical net benefits of CS dynamic nomogram at 1- year, 2-year, and 3-year. **(E) **C-index of the nomogram. **(F) **The CS dynamic nomogram calibration curves at 1, 2, and 3 years. **(G)** Time-dependent ROC curves estimate the nomogram’s forecasting value at 1, 2, and 3 years. ***p < 0.001.

After establishing the dynamic nomogram (**Figure 11A**), DCA was used to assess the net clinical benefit of the nomogram-integrated clinical features of the risk score in forecasting the probability of survival at 1, 2, and 3 years. As depicted in **Figure 11B-D**, the CS risk score and integrated nomogram exhibited similar clinical benefits in predicting 1-year survival and were superior to the benefits of other clinical features. Subsequent analyses demonstrated that the integrated nomogram had the highest clinical benefit in predicting survival at 2 and 3 years compared with other clinical features and was superior to the CS score alone and other parameters. The risk score and nomogram C-index were superior to any other independent factor (**Figure 11E**). The calibration curves further indicated that the survival outcomes of patients forecasted by the nomogram at 1, 2, and 3 years remained strikingly similar to the actual survival results (**Figure 11F**). Finally, we performed time-dependent ROC analysis to assess the predictive validity of the CS nomogram model. The findings demonstrated AUCs of 0.787, 0.718, and 0.733 for the prediction of 1-year, 2-year, and 3-year OS, respectively (**Figure 11G**). These findings suggest that the CS-score-based dynamic nomogram can accurately predict the prognosis of HBV-related HCC.

## Discussion

Chronic HBV infection is accompanied by insufficient immune surveillance (10, 41) and the induction of hepatocyte senescence (42), which leads to HCC. Thus, HBV represents a crucial link between cellular senescence and the development of HCC. The present study revealed the cellular senescence activation phenotype to have the following characteristics: 1. DNA damage response, telomere shortening, and activation of relevant pathways; 2. cyclin-dependent kinase inhibitors and cell cycle arrest; and 3. immune senescence. GSVA results further confirmed the reliability of our CS classification. Cell cycle-related signals, including G2M checkpoints, E2F targets, MYC targets, and the accumulation of cellular damage events, such as DNA repair and apoptosis, as well as mTOR signaling (43), were enriched in the C2 senescence-activated subtype. The mTOR signaling pathway is upregulated in senescent cells, activating the SASP phenotype, secreting inflammatory factors, promoting inflammation, and altering the immune microenvironment (44, 45). Senescent T cells exhibit robust glucose metabolism, while lipid metabolism is impeded (46), which is consistent with our GSVA results with more senescent T cell subpopulations in the C2 subgroup.

We demonstrated that cluster 2 contributed to higher levels of infiltration of dendritic cells, antigen-presenting cell (APC) co-inhibition, macrophages, MHC class 1, and T cell co-inhibition, whereas para-inflammatory components, B cells, active cytolytic components, and IFN response were distributed more in cluster 1 than in cluster 2. The C1 subtype resembles the T-cell inflammation-like phenotype, characterized by high expression of chemokines, T-cell markers, and IFN (47). Furthermore, innate immune recognition of cancer cells early *in vivo* involves activation of the type I IFN production pathway. Tumors can induce host APCs to produce type I IFNs, which in turn are necessary for the full activation of dendritic cells and initiation of spontaneous CD8+ T cells, and in turn, this leads to an immune phenotype of T cell infiltration (48). The immune landscape in C2 showed that the cellular senescence program was closely correlated with self-limited antitumor immunity and immune inhibition. T-cell infiltration has been linked to favorable clinical outcomes in HBV-related HCC (49–51). A recent study also showed that the number of cytotoxic T cells (CTLs) is reduced during the progressive phase of HBV-related HCC and is significantly associated with higher mortality and reduced survival time in HBV-related HCC (50). Furthermore, we revealed that NKT cells, which are critical for innate immunity (52), were negatively associated with the risk score and the five CS genes. Thus, differences in the immune composition between the two patterns may contribute to the poor prognosis of the C2 senescence-activated cluster. Therefore, cellular senescence could reprogram the immune cell composition of HBV-related HCC, thereby affecting its prognosis.

Subsequently, using combined univariate Cox and lasso multiple regression, five CSRGs (CENPA, EZH2, G6PD, PDAC1, and PRPF19) were employed to design the CS score signature. In two different cohorts, we verified these findings and noticed that patients with high-risk scores had a worse prognosis. Moreover, CSRGPI can be used as a reliable and independent prognostic predictor for HBV-related HCC. Recent reports demonstrated that the five crucial genes has important roles in HBV-related HCC. CENPA expression is upregulated in HBV-related HCC; CENPA serves as an oncogene in the progression of HBV-related HCC (53). High expression of the EZH2 in HCC accompanies tumor progression and the immunosuppressive microenvironment (54). Another study reported that EZH2 was negatively correlated with the IFN-gamma signaling pathway and positively correlated with the MYC and glycolytic signaling pathways, with respect to tumor growth and aggressiveness (55). Glucose-6-phosphate diphosphate (G6PD) catalyzes the pentose phosphate pathway, which controls oxidative stress and glucose metabolism (50). Liu et al.reported elevated G6PD expression levels in HBV-mediated liver cancer (56), and another study confirmed that G6PD knockdown suppressed hepatocarcinogenesis (57), further confirming the findings of this study. Previous studies have also demonstrated that HDAC1 modulates the senescence process of HCC cells (58), as well as that inhibiting HDAC1 expression induces apoptosis in tumor cells (59). PRPF19 is a predictive factor for worse clinical outcomes in HBV-related HCC and is associated with tumor immune evasion and progression (60). Moreover, WGCNA (47) analysis confirmed the correlation of CS score with cellular senescence signaling and oncogenic signaling, highlighting the need for the development of new immunotherapeutic agents.

It has demonstrated that the CS score model was significantly correlated with the immune microenvironment of HBV-related HCC. Malignant tumors may avoid immune destruction by activating immune checkpoint target genes (e.g., PD-1, PD-L1, CTLA-4, TGF-β, and HAVCR2). We found that the high-risk group showed a more uniform distribution of immune checkpoint expression. Similarly, the majority of immune checkpoint genes showed a favorable correlation with CS-based prediction models. These results suggest that the CS score may be a reliable biomarker for TME prediction and may be advantageous for ICI therapy forecasting.

**Figure 12:**
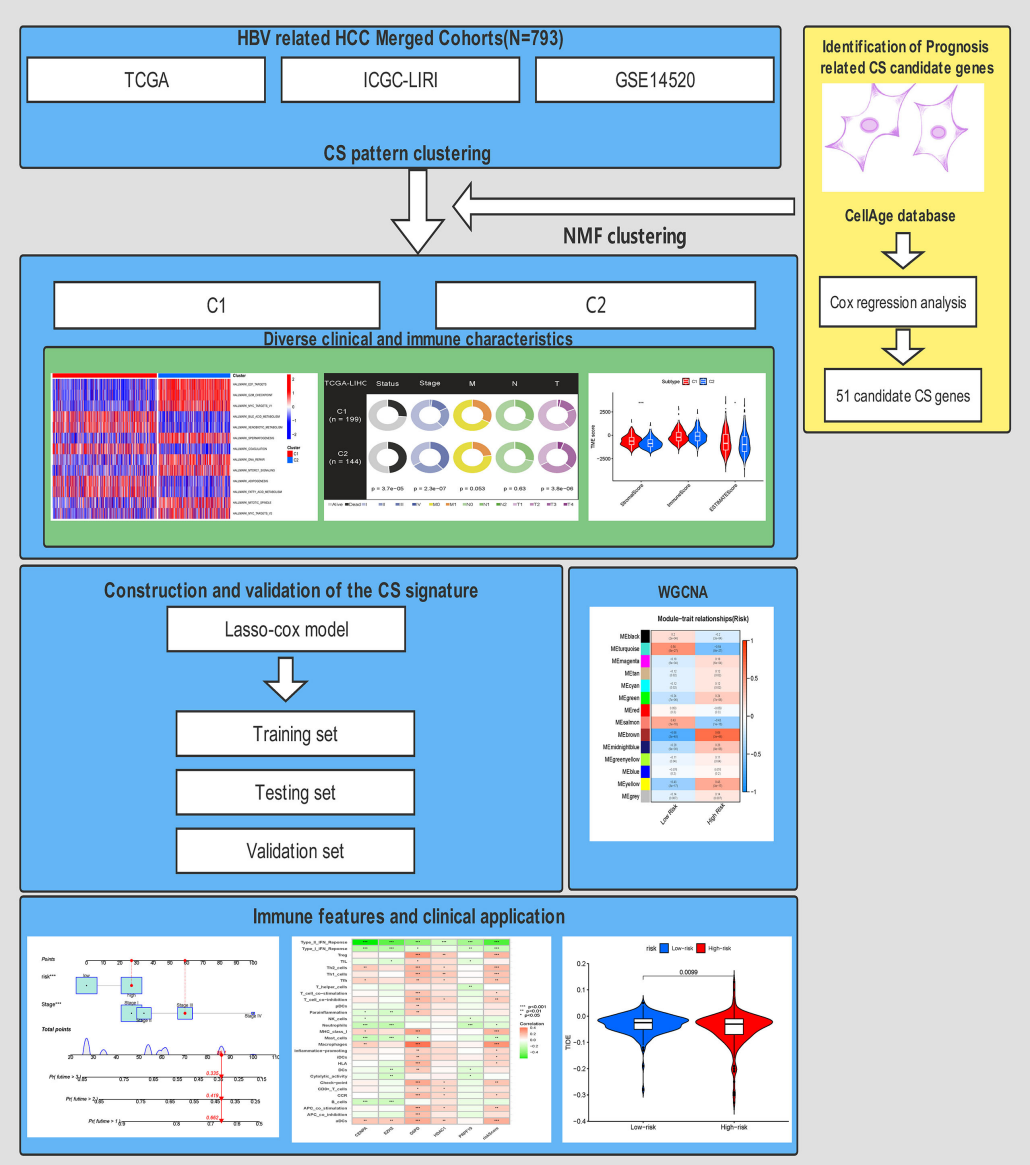
The workflow for this research.

Notably, in distinct clinical stratification, the CS score exhibited remarkable performance in distinguishing fatal survival outcomes. Finally, we generated a dynamic nomogram to predict the 1-, 3-, and 5-year survival probabilities of each patient by integrating the CS score and patient stage. We performed a series of tests to evaluate the discrimination and calibration capacities. The results revealed that the CS risk score is a reliable prognostic indicator to predict the prognosis of HBV-related HCC based on a CS dynamic nomogram. The workflow of this study is shown in **Figure 12**.

We acknowledge that this study has some limitations. First, the conclusions of the CS model were derived from analysis of a database that was assumed to be valid. Further panel validation should be conducted in a multicenter cohort of clinical patients with HCC in the future, to determine the value of CS scores in practical clinical applications, and further molecular biological validation of the CS score and the biological significance of critical genes in quantifying the senescent score should be designed. In future studies, we will further explore the aging-related aspects discussed herein.

## Data availability statement

The datasets presented in this study can be found in online repositories. The names of the repository/repositories and accession number(s) can be found in the article/**Supplementary Material**.

## Ethics statement

The data source involved in this study was open access; therefore, ethics approval was not applicable. Written informed consent for publication was obtained from all participants.

## Author contributions

XY and PC conceived the study and participated in the study design, performance, and manuscript writing. PC, WY, and WY conducted the bioinformatics analysis. XX supervised all the procedures and reviewed and revised this article. All authors read and approved the final manuscript. All authors approved the submitted version and indicated no conflict of interest. XX supervised all the procedures and reviewed and revised this article.

## Funding

This research was supported by the National Natural Science Foundation of China(81403434). The corresponding author XX, is a recipient of the National Natural Science Foundation of China(81403434).

## Conflict of interest

The authors declare that the research was conducted in the absence of any commercial or financial relationships that could be construed as a potential conflict of interest.

## Publisher’s note

All claims expressed in this article are solely those of the authors and do not necessarily represent those of their affiliated organizations, or those of the publisher, the editors and the reviewers. Any product that may be evaluated in this article, or claim that may be made by its manufacturer, is not guaranteed or endorsed by the publisher.

## Supplementary material

Supplementary Figure 1 The TIDE score between the different **(A)** CS patterns and risk groups **(B)**.

Supplementary Figure 2 The PCA down-dimension test exhibited distinct discrimination between different clusters in meta-cohorts. **(A) **The PCA analysis between the CS phenotypes. **(B) **The survival curve analysis of five CS-genes.

Supplementary Figure 3 The PCA down-dimension test exhibited distinct discrimination between different clusters in meta-cohorts. **(A)** The PCA analysis between the CS phenotypes. **(B)** The survival curve analysis of five CS-genes.

Supplementary Figure 4 GO analysis of co-expressed gene modules and correlation between CSRG risk score and the hallmark pathway. **(A)** GO enrichment analysis and classification based on the brown module were performed on three categories: CC, MF, and BP. **(B)** GO enrichment analysis and classification based on three categories of CC, MF and BP were performed on the turquoise module. **(C)** The correlation heat map of CS risk score and the hallmark pathway. **(D)** The correlation heat map of CS risk score and GSVA enrichment pathway.

## Abbreviations

CS, Cellular Senescent; TME, Tumor immune microenvironment; NMF, Non-negative Matrix Factorization; TCGA, The Cancer Genome Atlas; TIME, Tumor Immune microenvironment; GEO, Gene Expression Omnibus; ICGC, International Cancer Genome Consortium; CIBERSORT, Estimating relative subsets of RNA transcripts; WGCNA, Weighted gene co-expression network analysis; LASSO, Least absolute shrinkage and selection operator.
