# Supplementary figures and images for: Identification of cell senescence molecular subtypes in prediction of the prognosis and immunotherapy of hepatitis B virus-related hepatocellular carcinoma
Source: Front Immunol. 2022 Oct 6;13:1029872. doi: 10.3389/fimmu.2022.1029872 (PMC9582940; doi:10.3389/fimmu.2022.1029872)

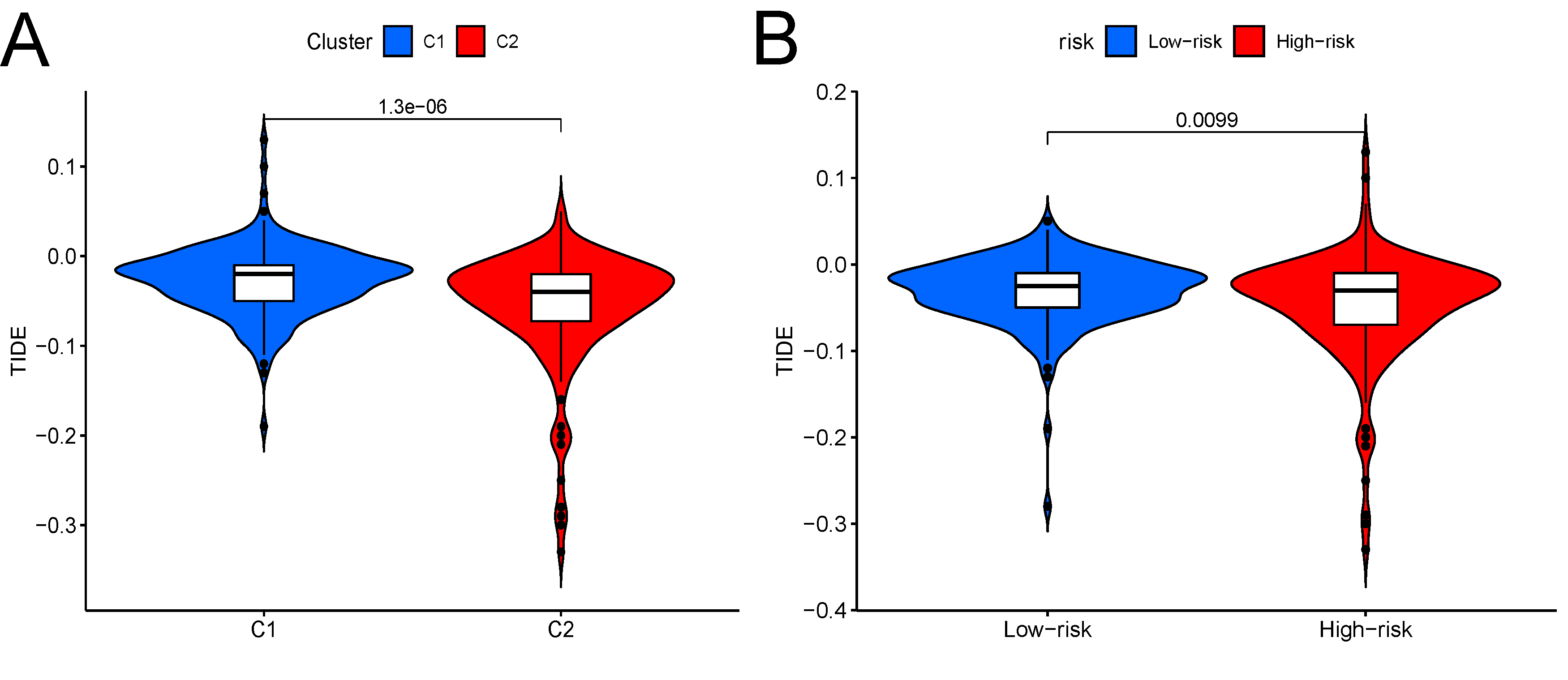

Supplement: Supplementary Figure 1 — The TIDE score between the different (A) CS patterns and risk groups (B). [file DataSheet_1.zip › Supplementary SFigure 1.tif]

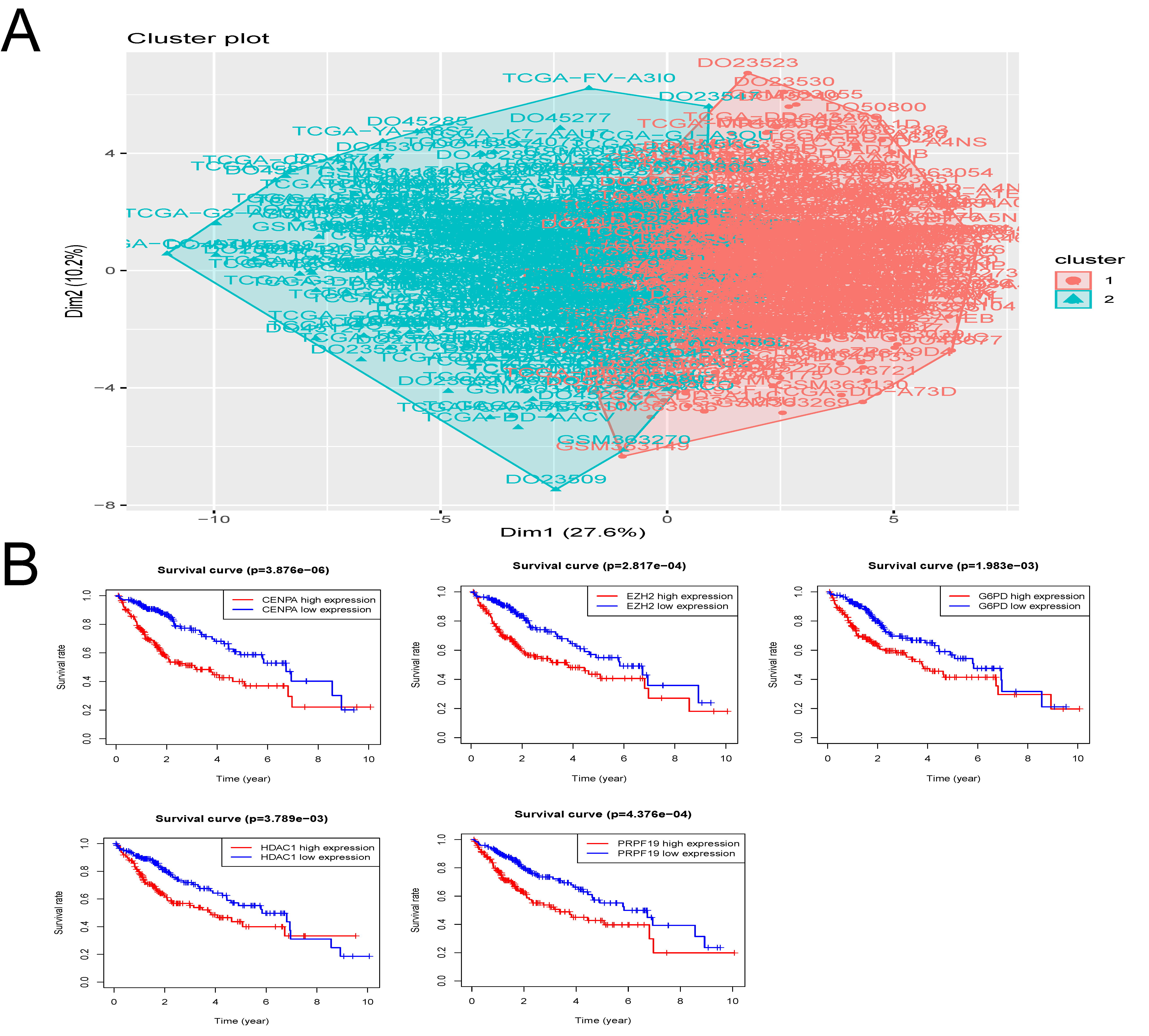

Supplement: Supplementary Figure 1 — The TIDE score between the different (A) CS patterns and risk groups (B). [file DataSheet_1.zip › Supplementary SFigure 2.tif]

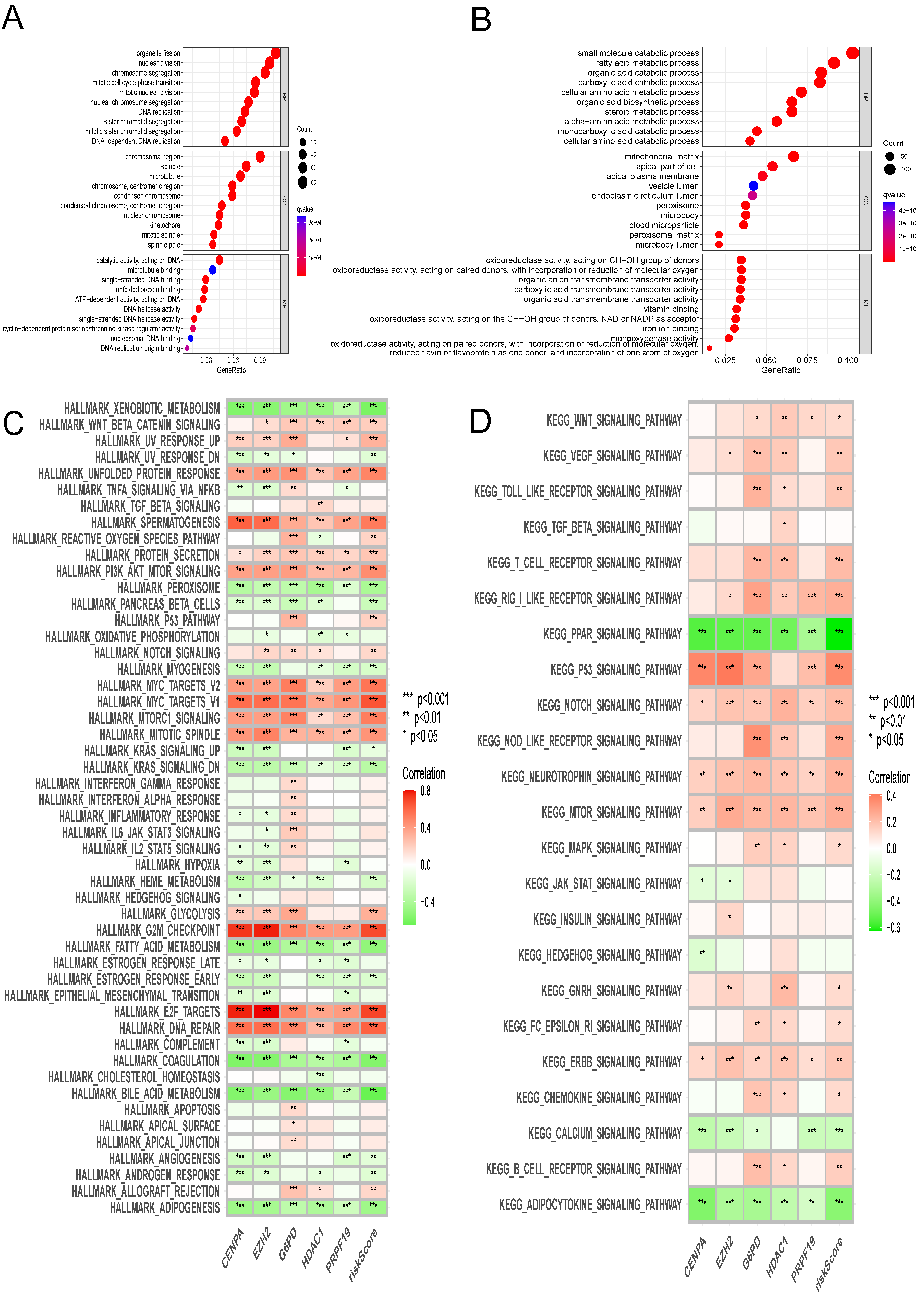

Supplement: Supplementary Figure 1 — The TIDE score between the different (A) CS patterns and risk groups (B). [file DataSheet_1.zip › Supplementary SFigure 3.tif]
